# Behavioural and metabolic mediators of socioeconomic inequalities in type 2 diabetes: comparing counterfactual and traditional mediation analysis

**DOI:** 10.1093/eurpub/ckaf056

**Published:** 2025-04-28

**Authors:** Diego Yacaman Mendez, Ylva Trolle Lagerros, Antonio Ponce de Leon, Per Tynelius, Stefan Fors, Anton Lager

**Affiliations:** Department of Global Public Health, Karolinska Institutet, Stockholm, Sweden; Centre for Epidemiology and Community Medicine (CES), Stockholm Health Care Services, Stockholm, Sweden; Centre for Obesity, Academic Specialist Centre, Stockholm Health Care Services, Stockholm, Sweden; Centre for Obesity, Academic Specialist Centre, Stockholm Health Care Services, Stockholm, Sweden; Clinical Epidemiology Division, Department of Medicine, Karolinska Institutet, Stockholm, Sweden; Centre for Epidemiology and Community Medicine (CES), Stockholm Health Care Services, Stockholm, Sweden; Department of Global Public Health, Karolinska Institutet, Stockholm, Sweden; Centre for Epidemiology and Community Medicine (CES), Stockholm Health Care Services, Stockholm, Sweden; Centre for Epidemiology and Community Medicine (CES), Stockholm Health Care Services, Stockholm, Sweden; Aging Research Centre, Karolinska Institutet and Stockholm University, Stockholm, Sweden; Department of Public Health Sciences, Stockholm University, Stockholm, Sweden; Department of Global Public Health, Karolinska Institutet, Stockholm, Sweden; Centre for Epidemiology and Community Medicine (CES), Stockholm Health Care Services, Stockholm, Sweden

## Abstract

There is a well-established social gradient in the occurrence of type 2 diabetes, but the extent to which behavioural or metabolic risk factors explain these inequalities remains unclear. Leveraging data from 7123 adults and over 20 years of follow-up, we used counterfactual mediation analysis to estimate the direct effect of low socioeconomic status (measured as educational attainment and occupational class) on the risk of type 2 diabetes, and the indirect effect through behavioural and metabolic risk factors. Mediators included were smoking, high alcohol consumption, low physical activity, diet low in vegetables or fruits, high body mass index (BMI), high fasting glucose, and hypertension. We compared the results to mediation analysis using the difference and the product of coefficients methods. We found an association between low educational attainment 1.31 (95% CI 1.16, 1.45) and low occupational class 1.24 (95% CI 1.09, 1.38) with future risk of type 2 diabetes. In the counterfactual mediation analysis, behavioural and metabolic risk factors explained 60% (95% CI 41%, 75%) of the effect of low educational attainment and 42% (95% CI 19%, 65%) of the effect of occupational class on the risk of type 2 diabetes. The difference and product of coefficients methods yielded similar results. Well-established behavioural and metabolic mediators explained roughly half of the health inequalities in the incidence of type 2 diabetes. Public health interventions should consider alternative mechanisms to reduce disparities in the incidence of type 2 diabetes.

## Introduction

Low socioeconomic status (SES) is an important risk factor for type 2 diabetes. Previous meta-analyses suggest a 30%–40% increased risk of type 2 diabetes among individuals exposed to low SES [1, 2]. Addressing these disparities in the incidence of type 2 diabetes is a pressing public health challenge.

The association between low SES and type 2 diabetes is complex. Partly, it can be explained by the higher prevalence of behavioural and metabolic risk factors and higher vulnerability to their negative effects among individuals exposed to low SES. In addition, the social determinants of health, i.e. ‘the conditions in which people are born, grow, live, work, and age’ [3] contribute to the association between SES and type 2 diabetes through other mechanisms such as influencing chronic stress levels, and shaping behavioural patterns of healthcare access and utilization [4, 5].

More evidence in needed on the potential equity implications of public health interventions at different levels. Guidelines for the prevention of type 2 diabetes have mainly focused on targeting individual-level lifestyle and metabolic risk factors [6]. More recently, there has been growing interest on the effects of economical and structural interventions to reduce socioeconomic disparities in the risk type 2 diabetes and its consequences [7]. However, the potential effect of these interventions remains uncertain.

Mediation analysis can be a useful method to estimate the effects of different pathways through which a low SES affects the risk of type 2 diabetes. Nevertheless, methodological challenges, such as exposure–mediator interactions or the simultaneous study of multiple mediators, hinder its use in complex associations. The use of counterfactual methods for mediation analysis allows to address some of these limitations, e.g. by controlling for exposure–mediator interactions [8]. However, the applicability of this framework in social epidemiology has been debated [9, 10].

In this study, we aimed to estimate the extent to which the association between low SES in adulthood and the incidence of type 2 diabetes is mediated through common metabolic and behavioural risk factors. In addition, we sought to compare the results of counterfactual and traditional mediation analyses.

## Methods

### Study sample

We used data from the Stockholm Diabetes Prevention Program (SDPP), a longitudinal cohort following healthy adults for the development of type 2 diabetes for over 20 years. The SDPP study was conducted in accordance with the ethical principles for medical research involving human subjects as outlined in the Declaration of Helsinki and was approved by the Ethics Committee of Region Stockholm (2019-06531).

Detailed information about SDPP is available elsewhere [11]. In summary, the entire registered population of five selected municipalities of Stockholm County, who were born in Sweden between 1938 and 1961, were invited to participate via post (32 368). A total of 26 717 (82.5%) agreed to participate and answered to a short questionnaire. From this larger sample, a clinical subsample was derived including all individuals with a positive family history of type 2 diabetes or previous gestational diabetes and matched controls which included 7948 individuals. A baseline examination was carried out in the clinical sample between 1992 and 1998, and follow-ups were conducted after approximately 10 and 20 years. Examinations included extensive health questionnaires, anthropometric and blood pressure measurements, and blood sample collections. Individual-level data were subsequently linked to population and healthcare registries using the Swedish personal identification number allowing us to follow-up all individuals for a new diagnosis of type 2 diabetes until 31 March 2021.

We excluded 128 individuals due to a diagnosis of type 2 diabetes during the baseline examination, 28 due to missing data on SES, 134 due to missing values of other covariates, 17 due to diagnosis of type 1 or LADA diabetes, 127 who emigrated from Sweden, and 393 due to death during the study period. The final study sample thus included data from 7123 individuals (4383 women and 2740 men), 89.6% of the baseline population.

### Variables

#### Diagnosis of type 2 diabetes

Incident cases of type 2 diabetes were ascertained from oral glucose tolerance tests, an inpatient or outpatient registered diagnosis of type 2 diabetes in the Stockholm Regional Healthcare Data Warehouse (VAL) or the National Diabetes Registry (NDR), or self-reported history of type 2 diabetes from the study questionnaires.

The oral glucose tolerance test (OGTT) was performed at each follow-up on all individuals without self-reported type 2 diabetes. A diagnosis of type 2 diabetes was made if fasting plasma glucose was ≥7.0 mmol/l or 2-h post load plasma glucose was ≥11.0 mmol/l [12]. From the VAL and NDR registries, the date of first diagnosis of type 2 diabetes was registered using the ICD-10 code E11. For self-reported diagnosis, individuals were asked if they had received a new diagnosis of type 2 diabetes by healthcare personnel.

#### Low SES

We used educational attainment and occupational class as measures of SES, using data from the longitudinal integrated database for health insurance and labour market (LISA). Educational attainment was assessed as the highest completed level of education with the following categories: basic education, vocational education or upper secondary education, and university education or higher. Low SES based on educational attainment was defined as a binary variable comparing individuals with educational attainment lower than upper secondary education to the rest. Occupational class was categorized according to the Swedish Socioeconomic Classification System into unskilled workers, semiskilled workers, skilled workers, assistant non-manual employees, employed professionals, self-employed professionals, and higher civil servants. We defined low SES based on occupational class as a binary variable including all unskilled and semiskilled manual workers.

#### Mediators

Our mediators were obtained during the baseline examinations and included well-established behavioural and metabolic risk factors for type 2 diabetes.

The behavioural risk factors included smoking, dichotomized as current smokers or previous/never smokers; alcohol consumption, estimated in centilitres per day from the study questionnaire and dichotomized as high alcohol consumption using the highest tercile, estimated for women and men separately; physical activity level, assessed with self-reports of physical activity compared to others of the same age, response options included much lower, lower, average, higher, and much higher and were categorized as low physical activity for those reporting very low or low physical activity; diet low in vegetables or fruits was assessed through a food frequency questionnaire (FFQ), including 49 food items for men and 54 for women, evaluated on a Likert scale with the options never or rarely, 1–2 times per month, 1 time per week, 2–3 times per week, 4–6 times per week, 1 time per day, 2–3 times per day, and >4 times per day. Low vegetable and fruit consumption was dichotomized to include participants who reported consumption of vegetables or fruits once per week or more seldom.

The metabolic risk factors included body mass index (BMI) calculated from measured weight and height, using height in metres divided by weight in kilograms squared, with obesity defined as a dichotomous variable including participants with a BMI ≥ 30 kg/m^2^; fasting glucose, measured in mmol/l from blood samples, with high fasting plasma glucose defined as a value ≥5.6 mmol/mol; and systolic and diastolic blood pressure, measured during the baseline examination in mm/Hg, and dichotomized as hypertension for values ≥140/90 mm/Hg or self-reported use of antihypertensive medications.

#### Confounders

Participants’ age at baseline, sex, family history of type 2 diabetes, self-reported comorbidities, and self-reported health status were included as confounders.

Age at baseline and sex were obtained from the Swedish general population register; family history of type 2 diabetes was self-reported and defined as at least one first- or two second-degree family members with type 2 diabetes; the presence of other chronic diseases was dichotomized and included all individuals who answered yes to the question ‘Do you have any chronic disease or injury after an accident?’. Self-reported health was gathered from the baseline questionnaire, and the answer options to the question ‘What is your general health status?’ were (1) very good, (2) good, (3) neither good nor bad, (4) bad, and (5) very bad. Answers 4 and 5 were dichotomized as ‘poor health’.

### Statistical analyses

The baseline characteristics of the study sample were summarized using mean and standard deviation (SD) for continuous variables and proportions for discrete values.

**Figure 1.**
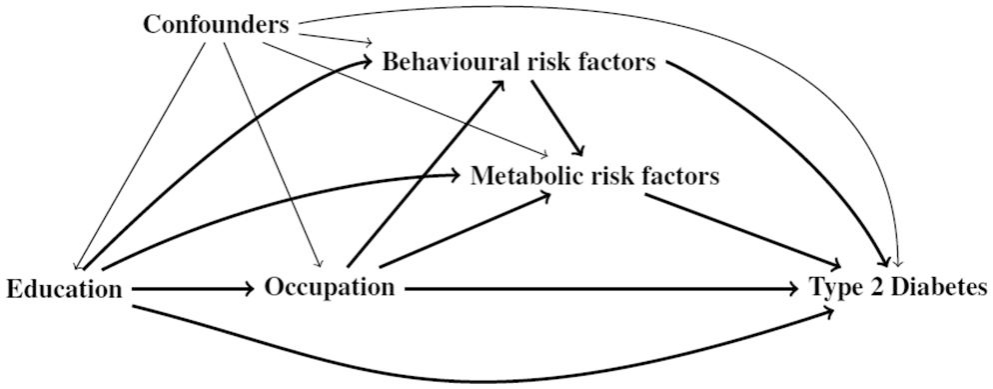
A directed acyclic graph (DAG) depicting the possible causal pathways linking low socioeconomic status to type 2 diabetes. The pathways include the effect of behavioural risk factors, metabolic risk factors, and confounders. Confounders included in our models included age, sex, self-reported health status, and comorbidities. The behavioural risk factors studied were smoking, alcohol consumption, low physical activity, and diet low in fruits or vegetables. Metabolic risk factors were high BMI, high fasting glucose, and hypertension.

For the counterfactual mediation analysis, we used modified Poisson regression to fit marginal structural model using inverse probability weights [8], based on the directed acyclic graph (DAG) shown in Fig. 1.

First, we obtained the stabilized inverse probability weights of exposure to low SES (*x*) adjusting for confounders (*c*):

$\mathrm{IPW}=\;\frac{P(x=1)}{P(x=1|c)}\;\mathrm{if}\;\mathrm{exposed},\;\mathrm{and}\;\mathrm{IPW}=\;\frac{1-P(x=1)}{1-P(x=1|c)}\;$ if unexposed.

Next, we used these to estimate the effect mediated through the mediators of interest taken together [natural indirect effect (NIE)], the effect through low SES [natural direct effect (NDE)], and the total effect (TE), according to the following equation [13]:

${\mathrm{TE}}_{\mathrm{RR}}={\mathrm{NIE}}_{\mathrm{RR}}\times {\mathrm{NDE}}_{\mathrm{RR}}=E\left[\frac{Y_{x}}{Y_{x^{*}}}\right]=\frac{E[Y_{xL_{x}M_{x}}]}{E[Y_{xL_{x^{*}}M_{x^{*}}}]}\times \frac{E\;[Y_{xL_{x^{*}}M_{x^{*}}}]}{E[Y_{x^{*}L_{x^{*}}M_{x^{*}}}]}$

where *L* are the behavioural risk factors, *M* is the metabolic risk factors, *x* is the exposure equal to 1 (i.e. low SES), and x* represents exposure set to 0 (i.e. middle, or high SES).

To estimate these counterfactuals, we used two regression models. First, a weighted Poisson regression of type 2 diabetes as outcome and low SES as exposure, to obtain the predicted average among exposed $E[Y_{x\;L_{x}M_{x}}]$ and unexposed ${E[Y}_{x^{*}L_{x^{*}}M_{x^{*}}}]$ individuals. Next, we used a weighted Poisson regression model with type 2 diabetes as the outcome and low SES, all mediators, confounders, and relevant exposure–mediator and mediator–mediator interactions as predictors to estimate the predicted probabilities of type 2 diabetes among unexposed $E[Y_{x\;L_{x^{*}}M_{x^{*}}}]$. As a sensitivity analysis, we estimated *E*-values for the association between low SES and type 2 diabetes and between the mediators and type 2 diabetes [14].

As comparison, we used the more conventional difference method and product of coefficient method for mediation analysis [15]. Both methods are based on the following regression models including the outcome (*Y*), exposure (*x*), and multiple confounders (*c*) and mediators (*m*), *p* represents the number of confounders and *q* the number of mediators [16]:


$$E\left[Y|x,c\right]=\alpha_{0}+\alpha x+\sum_{j=1}^{p}\theta_{j}c_{j}$$

$$E\left[Y|x,m,c\right]=\beta_{0}+\beta x+\sum_{j=1}^{p}\theta c_{j}+\sum_{j=1}^{q}\mu_{j}m_{j}$$

$$E\left[M_{i}|x,c\right]=\gamma_{0}+\gamma x+\sum_{j=1}^{p}\theta_{j}c_{j}$$

In the difference method, the total effect is given by the coefficient αx, the direct effect by the coefficient βx, and the indirect effect by their difference (αx-βx). In the product method, the total effect is given by the combination $\beta x+(\gamma x\;*\;\mu_{j}m_{j})$, the direct effect by the coefficient βx, and the indirect effect by the product of the coefficients $\gamma x\;*\;\mu_{j}m_{j}$. The total indirect effect through all mediators was estimated as the addition of the individual indirect effect of each mediator. Standardized coefficients were used to ensure comparability between models [15].

For all methods, the proportions mediated were estimated as the division of the indirect effect by the total effect. And 95% confidence intervals were obtained using bootstrap with 1000 reiterations.

The results were reported according to the AGReMA statement [17]. Analyses were done using Stata version 17 [18]. The code can be found in the Supplementary Materials.

## Results

A total of 1308 incident cases of type 2 diabetes were recorded with a clear social gradient observed in both the incidence of type 2 diabetes and the behavioural and metabolic mediators, as well as the confounders included in this study. Individuals with low SES, as defined by educational attainment or occupation, had a higher incidence of type 2 diabetes (25% vs 17% for education and 22% vs 17% for occupation) and a higher prevalence of all mediators and confounders assessed. The baseline characteristics of the study sample are present in Table 1 and in Supplementary Table S1.

**Table 1.** Distribution of confounders and mediators by exposure to measures of socioeconomic status

	Total	Education		Occupation	
	7123	Middle/high SES	Low SES	Middle/high SES	Low SES
Age (SD)	46.96 (4.94)	46.77 (4.96)	47.83 (4.73)	47.16 (4.90)	46.49 (5.00)
Women, *n* (%)	4383 (61.5%)	3655 (62.9%)	728 (55.4%)	3192 (63.4%)	1,191 (57.0%)
Men, *n* (%)	2740 (38.5%)	2153 (37.1%)	587 (44.6%)	1842 (36.6%)	898 (43.0%)
Family history of type 2 diabetes, *n* (%)	3741 (52.5%)	2981 (51.3%)	760 (57.8%)	2560 (50.9%)	1,181 (56.5%)
Comorbidities, *n* (%)	1958 (27.5%)	1567 (27.0%)	391 (29.7%)	1295 (25.7%)	663 (31.7%)
Self-rated poor general health, *n* (%)	143 (2.0%)	104 (1.8%)	39 (3.0%)	75 (1.5%)	68 (3.3%)
Current smoking, *n* (%)	1805 (25.3%)	1310 (22.6%)	495 (37.6%)	1112 (22.1%)	693 (33.2%)
High alcohol intake, *n* (%)	1380 (19.4%)	1099 (18.9%)	281 (21.4%)	992 (19.7%)	388 (18.6%)
Low physical activity, *n* (%)	1722 (24.2%)	1340 (23.1%)	382 (29.0%)	1152 (22.9%)	570 (27.3%)
Diet low in fruits or vegetables, *n* (%)	2920 (41.0%)	2314 (39.8%)	606 (46.1%)	2026 (40.2%)	894 (42.8%)
Body mass index (BMI) kg/m^2^ (SD)	25.58 (3.91)	25.41 (3.86)	26.33 (4.06)	25.34 (3.78)	26.15 (4.17)
BMI >30 kg/m^2^, *n* (%)	850 (11.9%)	626 (10.8%)	224 (17.0%)	525 (10.4%)	325 (15.6%)
Fasting plasma glucose mmol/l (SD)	4.71 (0.53)	4.68 (0.52)	4.81 (0.57)	4.69 (0.52)	4.75 (0.56)
Fasting plasma glucose >5.6 mmol/l, *n* (%)	360 (5.1%)	262 (4.5%)	98 (7.5%)	236 (4.7%)	124 (5.9%)
Systolic blood pressure (SBP) mmHg (SD)	122.40 (15.64)	121.84 (15.59)	124.88 (15.62)	122.04 (15.47)	123.29 (16.01)
Diastolic blood pressure (DBP) mmHg (SD)	76.81 (9.99)	76.52 (9.96)	78.10 (10.07)	76.62 (9.84)	77.28 (10.34)
Hypertension, *n* (%)	923 (13.0%)	722 (12.4%)	201 (15.3%)	621 (12.3%)	302 (14.5%)
Type 2 diabetes, *n* (%)	1308 (18.4%)	979 (16.9%)	239 (25.0%)	833 (16.5%)	475 (22.7%)

Data are presented as the mean and standard deviation (SD) for continuous measures and number of observations and proportions for categorical variables.

After adjusting for confounders, the incidence rate ratio (IRR) of type 2 diabetes for individuals exposed to low educational attainment was 1.29 (95% CI: 1.16, 1.43) compared to those with higher educational level. Further adjustment for behavioural and metabolic mediators resulted in a lower IRR of 1.10 (95% CI: 0.99, 1.20). For occupational status, adjusting for confounders, the IRR of type 2 diabetes was 1.23 (95% CI: 1.08, 1.40) for those with lower compared to higher occupational status. Additional adjustment for mediators attenuated the association to IRR 1.14 (95% CI: 1.02, 1.29). The complete results of the association between low SES and type 2 diabetes are present in Table 2 and the associations between measures of low SES, mediators, and type 2 diabetes are present in Supplementary Figs S1–S3.

**Table 2.** Logistic regression analyses for the association between measures of low socioeconomic status and type 2 diabetes in the SDPP study

	Model one IRR (95% CI)	Model two IRR (95% CI)	Model three IRR (95% CI)
Education	1.37 (1.23, 1.53)	1.29 (1.16, 1.43)	1.10 (0.99, 1.20)
Occupation	1.36 (1.23, 1.50)	1.23 (1.08, 1.40)	1.14 (1.02, 1.29)
Women			
Education	1.46 (1.24, 1.72)	1.36 (1.16, 1.60)	1.16 (0.98, 1.35)
Occupation	1.46 (1.26, 1.69)	1.30 (1.10, 1.54)	1.17 (0.99, 1.38)
Men			
Education	1.30 (1.13, 1.50)	1.23 (1.07, 1.42)	1.06 (0.92, 1.20)
Occupation	1.27 (1.11, 1.45)	1.17 (1.00, 1.38)	1.11 (0.94, 1.31)

All models were fitted using Poisson regression. IRR: incidence rate ration. 95% CI: 95% confidence intervals. Model one: adjusted for age at baseline and sex in the full cohort. Model two: adjusted for age at baseline, sex, comorbidities, family history of type 2 diabetes, and self-reported health, the occupation model was also adjusted for education. Model three: Model two and smoking, alcohol consumption, diet low in vegetables or fruits, physical activity, BMI ≥30 kg/m^2^, hyperglycaemia, and hypertension.

### Mediation analysis

#### Education

Using counterfactual mediation analysis, the relative risk (RR) of incident type 2 diabetes was 1.31 (95% CI: 1.16, 1.45) higher for low educational attainment compared to upper secondary education or higher. The RR for the direct effect was 1.12 (95% CI: 1.00, 1.24) and for indirect effect it was 1.17 (95% CI: 1.12, 1.22). The proportion of the total effect mediated through the studied behavioural and metabolic risk factors was 59.9% (95% CI: 41.4%, 75.1%).

Mediation using the difference method resulted in a total effect of 1.29 (95% CI: 1.16, 1.43), a direct effect of 1.09 (95% CI: 0.99, 1.20), an indirect effect of 1.18 (95% CI: 1.12, 1.25), and a proportion mediated of 65.8% (95% CI: 44.9%, 99.7%). The product of coefficients method indicated that low educational attainment was associated with a 1.28 (95% CI: 1.13, 1.43) increase in the relative risk of type 2 diabetes compared to upper secondary school education or higher. The direct effect was 1.09 (95% CI: 0.99, 1.20), and the indirect effect was 1.17 (95% CI: 1.10, 1.24). The proportion of the effect mediated was 63.0% (95% CI: 38.3%, 87.7%).

#### Occupational status

Counterfactual mediation analysis showed that the total effect of low occupational status was a RR of 1.24 (95% CI: 1.09, 1.38) higher risk of type 2 diabetes, compared to having an occupation categorized as medium or high SES. The RR for the direct effect was 1.13 (95% CI: 1.01, 1.26) and for the indirect effect was 1.09 (95% CI: 1.04, 1.15). Common behavioural and metabolic risk factors mediated 41.8% (95% CI: 19.1%, 65.5%) of the effect of low occupational status on the incidence of type 2 diabetes.

Using the difference method, we estimated a total effect of occupational status on the risk of type 2 diabetes of 1.28 (95% CI: 1.16, 1.42), which was formed by a direct effect of 1.16 (95% CI: 1.06, 1.28) and an indirect effect of 1.10 (95% CI: 1.06, 1.15), accounting for 39.6% (95% CI: 19.8%, 59.4%) of the association. With the product of coefficients methods, exposure to low occupational level was associated with a total effect of 1.24 relative increase in the risk of type 2 diabetes (95% CI: 1.10, 1.38) divided into a direct effect of 1.13 (95% CI: 1.02, 1.24) and an indirect effect through mediators of 1.09 (95% CI: 1.03, 1.16) compared to higher occupational categories. Behavioural and metabolic risk factors mediated 42.3% (95% CI: 22.6%, 69.4%) of the total effect. Comprehensive results of mediation analysis are present in Table 3.

**Table 3.** Mediation analysis using the counterfactual, difference, and the product of coefficients methods

	Education (*n* = 7123)			Occupation (*n* = 7123)		
	Counterfactual mediation	Difference method	Product of coefficients	Counterfactual mediation	Difference method	Product of coefficients
TE	1.31 (1.16, 1.45)	1.29 (1.16, 1.44)	1.28 (1.13, 1.43)	1.24 (1.09, 1.38)	1.28 (1.16, 1.42)	1.24 (1.10, 1.38)
NDE	1.12 (1.00, 1.24)	1.11 (1.00, 1.24)	1.09 (0.99, 1.20)	1.13 (1.01, 1.26)	1.16 (1.06, 1.28)	1.13 (1.02, 1.24)
NIE	1.17 (1.12, 1.22)	1.27 (1.12, 1.23)	1.17 (1.10, 1.24)	1.09 (1.04, 1.15)	1.10 (1.06, 1.15)	1.09 (1.03, 1.16)
PM	59.93% (41.43%, 75.08%)	59.65% (32.83%, 87.47%)	62.99% (38.31%, 87.66%)	41.76% (19.12%, 65.46%)	39.59% (19.76%, 59.41%)	42.34% (15.58%, 69.11%)
Women	Education (*n* = 4383)			Occupation (*n* = 4383)		
TE	1.38 (1.16, 1.63)	1.37 (1.16, 1.61)	1.38 (1.12, 1.63)	1.32 (1.11, 1.53)	1.30 (1.12, 1.51)	1.29 (1.08, 1.51)
NDE	1.14 (0.98, 1.35)	1.18 (1.01, 1.37)	1.16 (0.98, 1.35)	1.14 (0.98, 1.32)	1.18 (1.03, 1.37)	1.16 (1.00, 1.32)
NIE	1.21 (1.11, 1.31)	1.15 (1.07, 1.25)	1.18 (1.07, 1.30)	1.15 (1.07, 1.24)	1.10 (1.03, 1.18)	1.12 (1.01, 1.22)
PM	58.53% (30.52%, 83.99%)	46.03% (16.54%, 75.52%)	52.46% (21.98%, 82.95%)	51.48% (25.76%, 77.93%)	36.28% (8.75%, 63.82%)	43.76% (7.19%, 78.32%)
Men	Education (*n* = 2740)			Occupation (*n* = 2740)		
TE	1.24 (1.07, 1.42)	1.24 (1.07, 1.43)	1.21 (1.04, 1.39)	1.18 (1.00, 1.37)	1.22 (1.07, 1.40)	1.17 (1.01, 1.34)
NDE	1.08 (0.94, 1.23)	1.06 (0.92, 1.22)	1.06 (0.92, 1.20)	1.13 (0.97, 1.29)	1.12 (0.98, 1.27)	1.10 (0.97, 1.24)
NIE	1.15 (1.08, 1.22)	1.17 (1.09, 1.24)	1.15 (1.07, 1.23)	1.04 (0.98, 1.11)	1.09 (1.04, 1.15)	1.06 (0.99, 1.13)
PM	64.61% (36.19%, 93.73%)	71.95% (21.37%, 1.23%)	71.65% (33.95%, 109.36%)	26.87% (−9.81%, 66.77%)	44.46% (10.58%, 78.35%)	39.52% (−2.19%, 81.23%)

Table showing the estimates of the total effect (TE), natural direct effect (DNE), natural indirect effect (NIE), and the proportion mediated (PM). All 95% confidence intervals (95% CI), given in parentheses, were estimated using bootstrap analysis with 1000 repetitions.

#### Sensitivity analysis

Sensitivity analysis using *E*-values showed that an unobserved confounder between low educational attainment and type 2 diabetes equal to or higher than RR = 1.95 would suffice to make the total effect not significant. The presence of an unmeasured confounder between the mediators and type 2 diabetes associated with each mediator and the outcome with a risk ratio of at least 1.62 would make the indirect effect non-significant.

For occupational class, sensitivity analysis showed similar results. An unobserved confounder between low occupational class and type 2 diabetes of a magnitude of at least 1.92 in the risk ratio scale would be sufficient to explain away the overall association. And an unmeasured confounder between the mediator and the outcome of 1.49 or larger would be needed to make the indirect effect null (see also Supplementary Table S3).

## Discussion

Our findings indicate that established behavioural and metabolic risk factors, usual targets for public health interventions, explained roughly half of the effect of low SES on the development of type 2 diabetes. In this large Swedish cohort study, the results of counterfactual mediation analysis and more conventional methods for mediation analysis were similar.

### Comparison with previous studies

The increased risk of type 2 diabetes among adults with low educational attainment or occupational class found in this study is in line with previous literature [1, 5, 19, 20]. There are also previous studies looking into the mediators of this association. A recent meta-analysis used the difference method to estimate the contribution of lifestyle factors on the risk of chronic diseases and found that they accounted for between 20% and 30% of the inequalities [21]. Another study used the product of coefficients and found that smoking and physical activity accounted for approximately 30% of the association between education and type 2 diabetes [22]. More recently, a study using counterfactual mediation found that high BMI alone accounted for 45% of the association between education and type 2 diabetes [23]. However, these studies focused on one or few mediators. Our study adds to the literature by examining the joint effect of behavioural and metabolic factors.

Furthermore, few studies have compared the use of counterfactual mediation analysis and traditional methods in observational research [24]. We found very similar results using these two approaches. A benefit of counterfactual mediation analysis over traditional methods is that it allows for control of exposure–mediator interactions [25]. The fact that we did not find significant interactions between the measures of low SES and behavioural and metabolic mediators (Supplementary Table S2) could help explain these similar results, as previous studies have pointed out [26]. The difficulty of looking into multiple mediators is a shared limitation of both counterfactual and traditional mediation methods. Difficulties arise due to the interactions between mediators, which could lead to biased results by duplicating the effect of different pathways [13, 25]. The approach we used in this study addresses this by combining all mediators into a single indirect effect. Other approaches are available if the goal is to look at specific pathways or mediators [27].

### Strengths and limitations

The strengths of this study include the longitudinal design, long follow-up, large sample size, individual-level linkage to national and regional registries, and detailed information on plausible mediators. Furthermore, data for exposure to low SES, mediators, and outcome were gathered at different time points, limiting the risk of bias due to reverse causation.

The findings of this study should be interpreted considering its limitations. First, the clinical sample of the SDPP study includes an overrepresentation of individuals with family history of diabetes. However, previous studies have concluded that the baseline SDPP sample is representative of the whole population of Stockholm [28].

Regarding measures of SES, which is a complex construct involving different dimensions [29, 30], we chose measures of education and occupation to cover the effect of low SES through different social aspects and stages of life [31].

We used binary measures for all variables for simplicity, which likely reduced the true variability of the associations. Furthermore due to the difficulty to accurately assess self-reported behaviours, measurement error of behavioural risk factors may result in an underestimation of the mediated effect [32]. As an example, we categorized alcohol consumption based on the tertiles of self-reported use, which could provide a biased estimate of other more complex measures of hazardous alcohol use.

There are also methodological challenges related to mediation analysis. In order to study different pathways and multiple association, mediation analysis often requires large sample sizes to achieve adequate power. Therefore, despite the large sample size, the mediated effects presented might be underpowered, leading to increased risk of type 2 error. Unmeasured confounders, between exposure and outcome or between the mediators and the outcome, are another possible source of bias. Based on the results of the sensitivity analysis using the *E*-value, it is unlikely that the associations could be explained away by the effect of unobserved confounders.

Finally, the identification of causal effects using the counterfactual framework relies on the assumptions of positivity, conditional exchangeability, and consistency. The validity of the consistency assumption has been widely debated for social constructs such as socioeconomic position [9] and for some behavioural and metabolic exposures [33–35], under the argument that causal effects are not identifiable when exposures are not well defined (i.e. several versions of the exposure exist).

### Implications for future research and public health

Social inequalities remain an important public health challenge. In the case of type 2 diabetes, current prevention strategies primarily focus on reducing individual-level metabolic and behavioural risk factors and suggest to target vulnerable populations more directly to reduce inequalities [6]. However, our study indicates that even if the inequalities in these mediators were completely levelled, substantive inequalities would remain. Our results highlight the need to investigate different approaches to reduce health inequalities.

An alternative would be to directly address the broader social determinants of health by implementing interventions at a societal, policy, or community levels [7]. Although previous studies have lifted social policies and other system level interventions, such as economic support [36, 37], improved education [38], or access to the labour market [39], as measures to prevent and reduce inequalities in type 2 diabetes and other non-communicable diseases. Their real-life effects and practical applications remain uncertain. Further studies are needed in order to determine their utility in real-life situations.

## Supplementary Material

ckaf056_Supplementary_Data

## Data Availability

The SDPP study is managed by the Centre for Epidemiology and Community Medicine (CES). Data from SDPP used in this article can be shared on reasonable request to the corresponding author in accordance to the General Data Protection Regulations of the European Union (GDPR). Key points
*What is already known on this topic*: There is a social gradient in the risk of type 2 diabetes, with individuals with low socioeconomic status being disproportionately affected. Reducing these inequalities is an important public health issue.
*What this study adds*: Prevention of type 2 diabetes is based on individual-level reduction of behavioural and metabolic risk factors. However, whether these interventions reduce inequalities in the risk of type 2 diabetes remains debated. We explore the potential effect of eliminating common risk factors in reducing social inequalities in the occurrence of type 2 diabetes.
*How this study might affect research, practice, or policy*: While addressing individual-level risk factors is important, it is unlikely to eliminate social inequalities in diabetes. Policymakers and public health practitioners should consider broader social interventions. A comprehensive approach that integrates both individual and structural interventions is essential to achieving health equity.
