# Supplementary material for: Behavioural and metabolic mediators of socioeconomic inequalities in type 2 diabetes: comparing counterfactual and traditional mediation analysis
Source: Eur J Public Health. 2025 Apr 28;35(4):605–10. doi: 10.1093/eurpub/ckaf056 (PMC12311358; doi:10.1093/eurpub/ckaf056)
Supplement: ckaf056_Supplementary_Data [file ckaf056_supplementary_data.docx]

Behavioural and metabolic mediators of socioeconomic inequalities in type 2 diabetes: comparing counterfactual and traditional mediation analysis

supplementary material

Diego Yacaman Mendez^a,b,c^, Ylva Trolle Lagerros^c,d^, Antonio Ponce de Leon^b^, Per Tynelius^a,b^, Stefan Fors^b,e,f^, Anton Lager^a,b^

^a^ Department of Global Public Health, Karolinska Institutet, Stockholm, Sweden

^b^ Centre for Epidemiology and Community Medicine (CES), Region Stockholm, Stockholm, Sweden

^c^ Centre for Obesity, Academic Specialist Centre, Region Stockholm, Stockholm, Sweden

^d^ Clinical Epidemiology Division, Department of Medicine, Karolinska Institutet, Stockholm, Sweden

^e^ Aging Research Centre, Karolinska Institutet and Stockholm University, Stockholm, Sweden

^f^ Department of Public Health Sciences, Stockholm University, Stockholm, Sweden

Correspondence to: Diego Yacaman Mendez M.D., Ph.D.

Department of Global Public Health, Karolinska Institutet, SE-171 77 Stockholm, Sweden

E-mail address: [diego.yacaman.mendez@ki.se](mailto:diego.yacaman.mendez@ki.se)

# Table S1. Baseline characteristics of the study sample by sex

|  | **Total** | **Women** | **Men** |
| --- | --- | --- | --- |
|  | **N=7,123** | **N=4,383** | **N=2,740** |
| **Low occupational status** | 2,089 (29.3%) | 1,191 (27.2%) | 898 (32.8%) |
| **Low educational attainment** | 1,315 (18.5%) | 728 (16.6%) | 587 (21.4%) |
| **Current smoking** | 1,805 (25.3%) | 1,137 (25.9%) | 668 (24.4%) |
| **High alcohol intake** | 1,380 (19.4%) | 870 (19.8%) | 510 (18.6%) |
| **Low physical activity** | 1,722 (24.2%) | 1,139 (26.0%) | 583 (21.3%) |
| **Diet low in fruits or vegetables** | 2,920 (41.0%) | 1,389 (31.7%) | 1,531 (55.9%) |
| **Body mass index (BMI) kg/m2 (SD)** | 25.58 (3.91) | 25.33 (4.18) | 25.98 (3.40) |
| **BMI >30 kg/m2** | 850 (11.9%) | 542 (12.4%) | 308 (11.2%) |
| **Fasting plasma glucose mmol/L (SD)** | 4.71 (0.53) | 4.70 (0.49) | 4.72 (0.60) |
| **Fasting plasma glucose >5.6 mmol/L** | 360 (5.1%) | 169 (3.9%) | 191 (7.0%) |
| **Systolic blood pressure (SBP) mmHg (SD)** | 122.4 (15.6) | 121.0 (16.2) | 124.6 (14.4) |
| **Diastolic blood pressure (DBP) mmHg (SD)** | 76.8 (10.0) | 74.9 (9.8) | 79.8 (9.6) |
| **Hypertension** | 923 (13.0%) | 524 (12.0%) | 399 (14.6%) |
| **Age (SD)** | 46.96 (4.94) | 47.26 (4.93) | 46.48 (4.91) |
| **Family history of type 2 diabetes** | 3,741 (52.5%) | 2,329 (53.1%) | 1,412 (51.5%) |
| **Self-reported comorbidities** | 1,958 (27.5%) | 1,250 (28.5%) | 708 (25.8%) |
| **Self-reported poor general health** | 23.51 (4.68) | 22.64 (3.79) | 24.91 (5.56) |
| **Incident cases of type 2 diabetes** | 1,308 (18.4%) | 636 (14.5%) | 672 (24.5%) |
| Data are presented as mean and standard deviation (SD) for continuous measures and number of observations and proportions for categorical variables. | | | |

# Table S2. Exposure-mediator interactions by measures of socioeconomic status

| **IRR of type 2 diabetes** | **Low educational attainment**  **(IRR)** | **Low occupational status**  **(IRR)** |
| --- | --- | --- |
| **Current smoking** | 0.85 (0.65, 1.02) | 0.75 (0.61, 0.93) |
| **High alcohol intake** | 0.95 (0.74, 1.23) | 0.88 (0.69, 1.13) |
| **Low physical activity** | 0.93 (0.75, 1.17) | 0.95 (0.77, 1.18) |
| **Diet low in fruits or vegetables** | 0.85 (0.71, 1.08) | 1.03 (0.85, 1.25) |
| **BMI >30 kg/m^2^** | 0.73 (0.59, 0.91) | 0.85 (0.70, 1.03) |
| **Fasting plasma glucose >5.6 mmol/L** | 0.65 (0.52, 0.81) | 0.78 (0.63, 0.96) |
| **Hypertension** | 0.87 (0.70, 1.09) | 0.96 (0.78, 1.19) |

Incidence risk ratios (IRR) of the interaction term between behavioural and metabolic mediators and measures of socioeconomic status. Estimates from modified Poisson regression models for incidence of type 2 diabetes, in addition to mediators and interaction term, the models were adjusted for age at baseline, sex, comorbidities, family history of type 2 diabetes and self-reported health.

# Table S3. Sensitivity analysis: E-values for the total effect and indirect effects.

|  |  | **Education** | | **Occupation** | |
| --- | --- | --- | --- | --- | --- |
|  |  | Total effect | Natural indirect effect | Total effect | Natural indirect effect |
| **Total** | Point estimate | 1.95 | 1.62 | 1.9 | 1.46 |
|  | Confidence interval | 1.62 | 1.46 | 1.62 | 1.34 |
| **Women** | Point estimate | 2.08 | 1.71 | 1.79 | 1.57 |
|  | Confidence interval | 1.59 | 1.46 | 1.34 | 1.37 |
| **Men** | Point estimate | 1.79 | 1.57 | 1.79 | 1.57 |
|  | Confidence interval | 1.34 | 1.37 | 1.34 | 1.37 |

E-values are given for the point estimates and the lower bound of the 95 confidence intervals. They represent the magnitude that an unmeasured confounder must have, in the risk ratio scale, to make the point estimate or lower bound of the confidence intervals null, respectively.

# Figure S1. Associations between measures of socioeconomic status, behavioural and metabolic mediators, and incidence of type 2 diabetes.


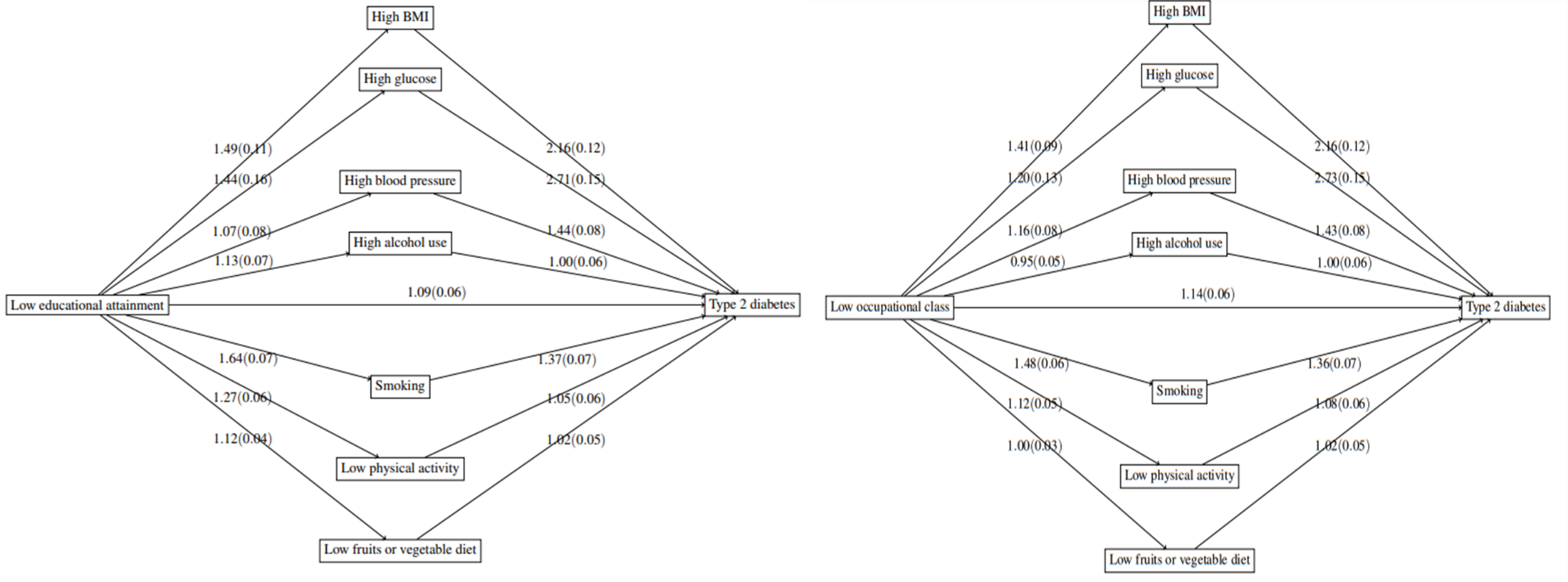


The results from Poisson regression models adjusted for sex, age, self-reported health, and comorbidities were used to estimate the effects. Point estimates are presented as incidence risk ratios (IRRs) and standard errors in parentheses.

# Figure S2. Associations between measures of low socioeconomic status, behavioural and metabolic mediators and incidence of type 2 diabetes stratified among women.


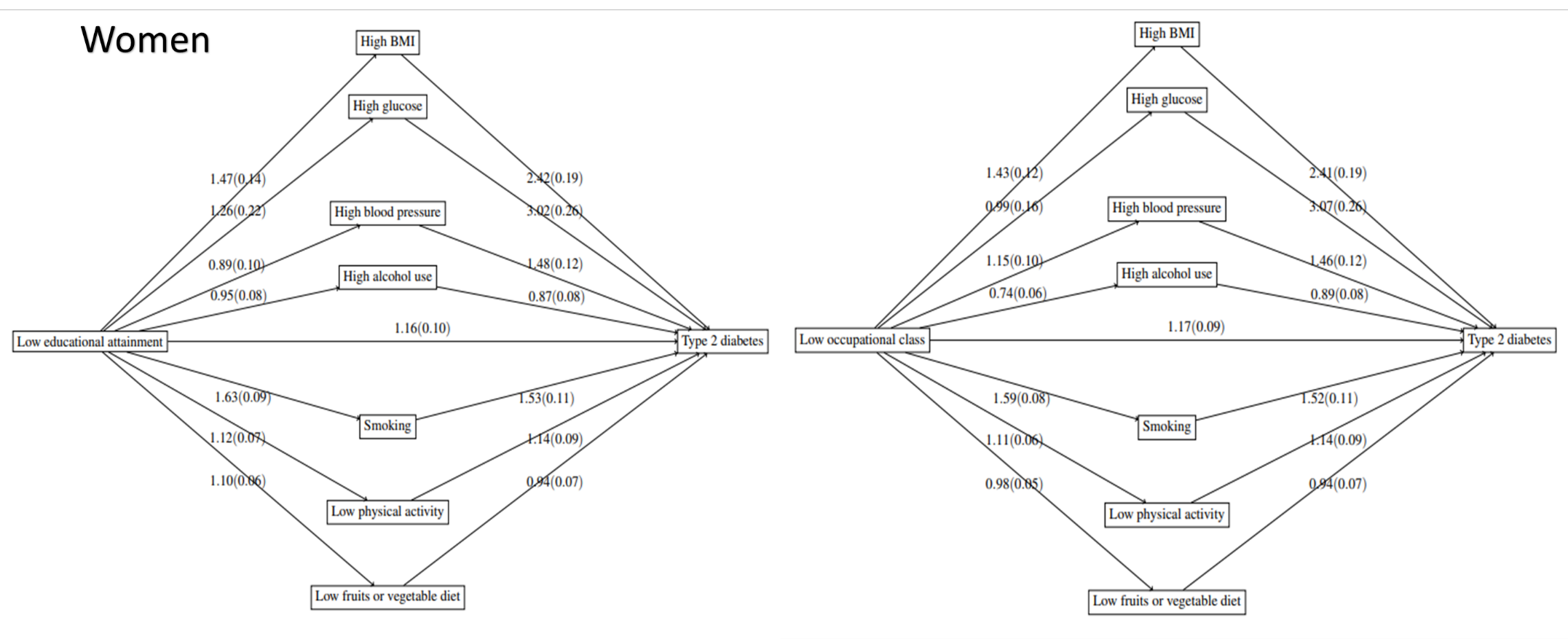


Poisson regression models adjusting for sex, age, self-reported health, and comorbidities were used to estimate the effects. Point estimates presented as incidence risk ratios (IRR) and standard errors in parentheses.

# Figure S3. Associations between measures of low socioeconomic status, behavioural and metabolic mediators and incidence of type 2 diabetes stratified among men.


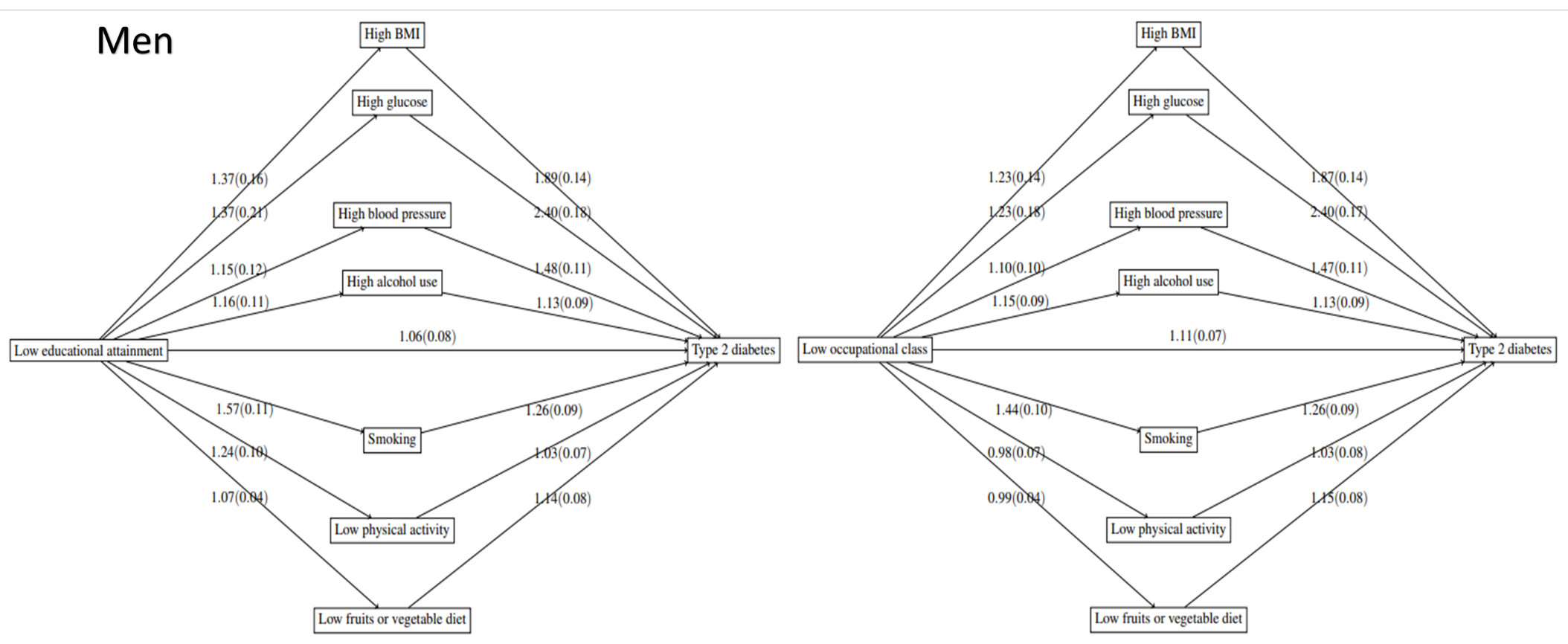


Poisson regression models adjusting for sex, age, self-reported health, and comorbidities were used to estimate the effects. Point estimates presented as incidence risk ratios (IRR) and standard errors in parentheses.

# Stata code

***************************************************************

// Generate a simulated dataset

* Note: the data is simulated randomly as an example, results will not match those presented in the manuscript.

***************************************************************

drop _all

set obs 10000

gen c = rbinomial(1, 0.3)

gen x = rbinomial(1, invlogit(logit(0.4)+ln(3)*c))

gen m1= rbinomial(1, invlogit(logit(0.4)+ln(2)*c+ln(4)*x))

gen m2= rbinomial(1, invlogit(logit(0.4)+ln(2)*c+ln(2)*m1+ln(4)*x))

gen y= rbinomial(1, invlogit(logit(0.4)+ln(2)*x+ln(2)*c+ln(10)*m1+ln(10)*m2))

***************************************************************

// Difference Method

***************************************************************

*Fit equations

poisson y x c,

estimates store mod1

poisson y x c m1 m2 ,

estimates store mod2

*Combine coefficients

suest mod1 mod2 , vce(robust) eform(RR)

*Estimate effects

*Total effect

nlcom([mod1_y]x), eform

*Direct Effect

nlcom([mod2_y]x), eform

* Indirect effect

nlcom ([mod1_y]x - [mod2_y]x), eform

* Proportion mediated

nlcom ([mod1_y]x- [mod2_y]x) / ([mod1_y]x)

***************************************************************

// Product Method

***************************************************************

*Fit equations

poisson m1 m2 x c ,

estimates store mod3

poisson m2 m1 x c ,

estimates store mod4

*Combine equations (needs mod2, see difference method)

suest mod2 mod3 mod4 , vce(robust) eform(RR)

*Estimate effects

*Total effect

nlcom ([mod2_y]x)+(([mod3_m1]x *[mod2_y]m1)+([mod4_m2]x *[mod2_y]m2)), eform

*Direct effect

nlcom([mod2_y]x), eform

* Indirect effect

nlcom ([mod3_m1]x *[mod2_y]m1)+([mod4_m2]x *[mod2_y]m2), eform

* Proportion mediated

nlcom (([mod3_m1]x *[mod2_y]m1)+([mod4_m2]x *[mod2_y]m2)) / (([mod2_y]x)+(([mod3_m1]x *[mod2_y]m1)+([mod4_m2]x *[mod2_y]m2))),

***************************************************************

// Counterfactual Mediation analysis (point estimates)

***************************************************************

*Estimate the inverse probability weights (IPWs)

poisson x, vce(robust)

predict pa,

poisson x c, vce(robust) irr

predict pac,

gen ipwa= pa/pac

replace ipwa= ((1-pa)/(1-pac)) if x==0

sum ipwa

**Estimate counterfactual Q1= E(Ya=0 Ma=0)

regress y [pweight=ipwa] if x==0

gen q1= _b[_cons]

**Estimate counterfactual Q2 E(Ya=1 Ma=1)

regress y [pweight=ipwa] if x==1

gen q2= _b[_cons]

** Estimate counterfactual Q3 E[Y|a,c,m]

poisson y x c m1 m2 c.x#c.m1 c.x#c.m2 c.m1#c.m2 , vce(robust) irr

**Q3 E(Ya=1 Ma=0)

margins, at(x=1) gen (qr3)

regress qr3 [pweight=ipwa] if x==0

gen q3= _b[_cons]

* Total Effect

di (q3-q1)+(q2-q3)

di (q3/q1)*(q2/q3)

*Direct Effect

di (q3-q1)

di (q3/q1)

*Indirect Effect

di (q2-q3)

di (q2/q3)

*Proportion Mediated

di (q2-q3)/((q3-q1)+(q2-q3))
